# Hydralazine-Induced Antineutrophil Cytoplasmic Antibody Vasculitis Causing Gastroduodenal Artery Perforation

**DOI:** 10.7759/cureus.106151

**Published:** 2026-03-30

**Authors:** Katherine N Gonzalez, Daniel Faradji, Kyle Schneider, Shivani Desai, Huda N Khan, Jaimin Patel

**Affiliations:** 1 Internal Medicine, Methodist Health System, Dallas, USA; 2 Internal Medicine, Methodist Dallas Medical Center, Dallas, USA

**Keywords:** anca-vasculitis, gastroduodenal artery perforation, gastrointestinal hemorrhage, hemoptysis, hydralazine

## Abstract

Antineutrophil cytoplasmic antibody (ANCA)-associated vasculitides are rare disorders characterized by inflammation of small blood vessels, including capillaries, venules, and arterioles. The three main subtypes of primary ANCA-associated vasculitides (i.e., granulomatosis with polyangiitis, microscopic polyangiitis, and eosinophilic granulomatosis with polyangiitis) can present with diverse and nonspecific symptoms, making diagnosis difficult. We report the case of secondary, drug-induced ANCA vasculitis in a female patient who presented with hemoptysis and dyspnea complicated by acute kidney injury and severe anemia. Serologic testing and renal biopsy confirmed the diagnosis of ANCA-associated vasculitis (AAV). Her hospital course was complicated by multiple gastrointestinal bleeds requiring two interventional radiology procedures, first on her gastroduodenal artery and later on her inferior pancreaticoduodenal artery. The patient was treated with cyclophosphamide and plasma exchange during hospitalization and was transitioned to rituximab and intravenous steroids, resulting in an eventual recovery. This case highlights the intricacies of diagnosing AAV and the potential severe, life-threatening complications, such as gastrointestinal hemorrhage. Early recognition and a multidisciplinary approach are critical to evaluate and treat as well as to improve outcomes in patients with this rare but serious condition.

## Introduction

Hydralazine is a vasodilator used to treat hypertension. Although it is no longer recommended as a first-line treatment, it continues to be used as a primary or adjunctive agent in certain patient populations. One rare but serious adverse effect of hydralazine therapy is the development of antineutrophil cytoplasmic antibody (ANCA)-associated vasculitis (AAV). AAV is a rare autoimmune disease characterized by inflammatory cell infiltration, causing necrosis of blood vessels [1]. The most common primary AAVs are granulomatosis with polyangiitis, microscopic polyangiitis, eosinophilic granulomatosis with polyangiitis, and drug-induced vasculitis [2]. The pathogenesis of drug-induced vasculitis is poorly understood and is thought to be related to an immune system reaction to a drug [3]. AAV can cause diverse and nonspecific symptoms, such as fever, night sweats, fatigue, anorexia, weight loss, arthralgias, and arthritis, making diagnosis difficult [4]. Diagnosis of drug-induced vasculitis is based on clinically evident vasculitis and administration of the offending drugs, such as hydralazine [5].

Hydralazine-induced AAV most commonly involves the kidneys and respiratory tract, presenting with clinical features such as hematuria, renal failure, hemoptysis, or pulmonary infiltrates [1-6]. Gastrointestinal involvement, however, is rare and not well characterized in the literature. We present the case of a 52-year-old female who developed hydralazine-induced AAV complicated by a retroperitoneal hematoma with gastroduodenal and pancreaticoduodenal artery involvement.

## Case presentation

A 52-year-old female with a medical history of anemia, nonischemic cardiomyopathy with implantable cardioverter-defibrillator placement, type 2 diabetes mellitus, hypertension, and hyperlipidemia presented to the emergency department with a two-day history of hemoptysis, dyspnea, chills, throat pain, and melena. The patient was hemodynamically stable and maintained oxygen saturation on room air. Physical examination was notable for blood-tinged sputum without clots. The patient reported a 20-pound weight loss over the preceding month. She was taking apixaban for a history of left upper extremity deep vein thrombosis (DVT) diagnosed in 2016, with her previous dose taken the day before presentation. Notably, the patient was also taking hydralazine 100 mg three times daily for hypertension for at least two years.

On admission, laboratory studies revealed a hemoglobin level of 5.7 g/dL and creatinine of 3.1 mg/dL (baseline: 1.1 mg/dL one year prior). Urinalysis showed proteinuria (urine protein: 161 mg/dL; protein-to-creatinine ratio: 2). The patient was transfused to maintain a hemoglobin goal of >7 g/dL, though transfusion was delayed due to crossmatch antibodies. Nephrology was consulted for the management of acute kidney injury with acidosis. A urine antigen test was positive for *Streptococcus pneumoniae*, and a noncontrast chest CT scan showed multifocal ground-glass opacities with upper lobe predominance. She was treated with azithromycin and ceftriaxone for *S. pneumoniae* pneumonia while awaiting autoimmune serologies, including antinuclear antibody (ANA), ANCA, and anti-glomerular basement membrane (anti-GBM) antibodies. Given her hemoptysis with acute kidney injury, there was a high suspicion for AAV. A lung perfusion study showed normal pulmonary perfusion, ruling out a pulmonary embolus.

**Figure 1:**
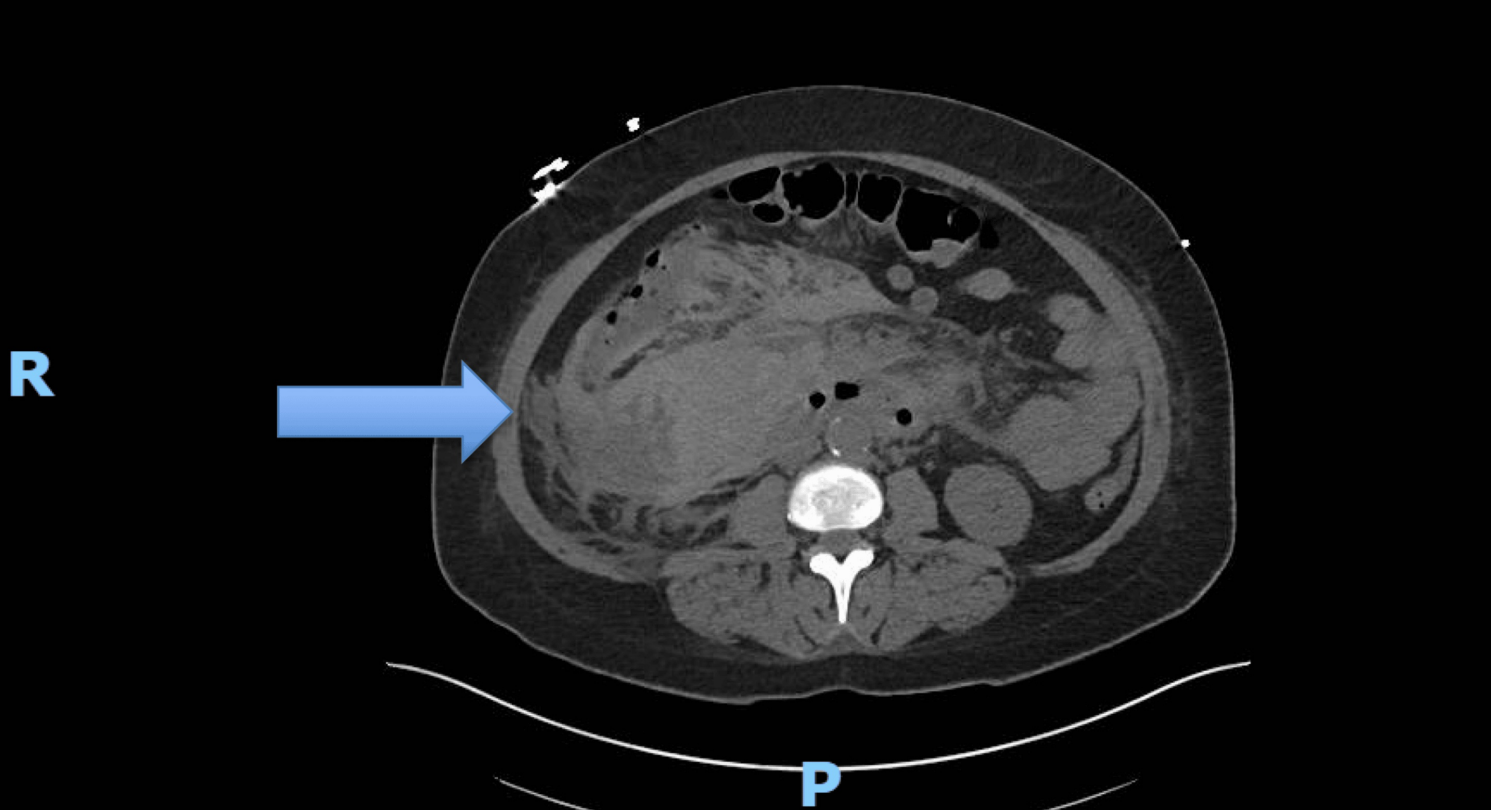
Axial CT image of the abdomen showing a large right retroperitoneal hematoma. The blue arrow indicates hematoma.

Despite appropriate antibiotic treatment for *S. pneumoniae*, hemoptysis persisted. On hospital day three, she developed sharp abdominal pain, hypoxia requiring a 2 L nasal cannula, and hypotension (blood pressure: 81/53 mmHg), prompting transfer to the intensive care unit. CT imaging of the abdomen revealed a large right retroperitoneal hematoma suspicious for duodenal ulcer perforation and a small perinephric hematoma (Figure 1). General surgery, interventional radiology (IR), and gastroenterology were consulted. The patient underwent successful gastroduodenal artery embolization and remained hemodynamically stable after the embolization without requiring further blood transfusions.

Autoimmune testing revealed elevated ANA levels with a titer of 1:320, positive p-ANCA, elevated myeloperoxidase (MPO) IgG, proteinase 3 (PR3) antibody, and anti-double-stranded DNA. See Table 1 for exact values and reference ranges.

**Table 1:** Selected relevant autoimmune laboratory values. ANA = antinuclear antibody; ANCA = antineutrophil cytoplasmic antibody; MPO = myeloperoxidase; PR3 = proteinase 3

Test	Result	Reference range	Interpretation
ANA level	1.59 IU/mL	<1.1 IU/mL	Elevated
ANA titer	1:320	≤1:40	Positive
ANCA	>1:1,280	<1:20	Elevated
MPO IgG	5.42 IU/mL	<1.1 IU/mL	Elevated
PR3 antibody	1.84 IU/mL	<1.1 IU/mL	Elevated
Anti–dsDNA	1.19 IU/mL	<1.1 IU/mL	Elevated
Anti–Jo-1 antibody	9 AU/mL	≤29 AU/mL	Negative
C4	17 mg/dL	14–44 mg/dL	Normal
C3	85 mg/dL	88–165 mg/dL	Mildly decreased

**Figure 2:**
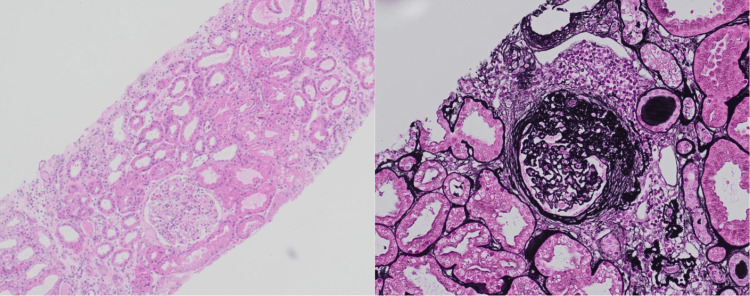
Kidney core needle biopsy images showing pauci-immune necrotizing glomerulonephritis with 40% cellular crescents and segmental glomerular scarring, moderate interstitial fibrosis, and tubular atrophy (30-40%) with associated acute tubular damage. The figure on the right shows a close-up of the glomerulus.

Given her positive p-ANCA as well as elevated MPO and PR3, she was suspected to have hydralazine-induced AAV. A renal biopsy showed pauci-immune necrotizing glomerulonephritis with 40% cellular crescents, segmental glomerular scarring, moderate interstitial fibrosis and tubular atrophy (30-40%), acute tubular damage, and marked arterial intimal hyperplasia (Figure 2).

Hematology and oncology were consulted for plasma exchange and cyclophosphamide. After five sessions, she developed hot flashes, diarrhea, and abdominal pain, followed by tachycardia, hypotension, and respiratory failure requiring intubation and vasopressor support. Laboratory evaluation showed a lactate of 11 mmol/L and hemoglobin of 3.4 g/dL, and she was transfused with four units of blood. CT angiography showed a pseudoaneurysm or active extravasation from the branch of the pancreaticoduodenal artery, a large infarct of the left hepatic lobe, small-volume hemoperitoneum, and a stable right retroperitoneal hematoma measuring 12.8 cm. Repeat IR embolization of the inferior pancreaticoduodenal artery was performed.

After discussion with general surgery and nephrology, it was determined that her gastrointestinal bleeding was related to her AAV, likely related to vasculitic involvement of two vascular beds. She was treated with pulse-dose intravenous methylprednisolone, followed by clinical stabilization, extubation, and transfer from the intensive care unit. She completed plasma exchange and was started on rituximab. The patient eventually was transitioned to a regular diet and was discharged to a skilled nursing facility on a steroid taper with planned outpatient follow-up for continued cyclophosphamide and rituximab therapy.

## Discussion

This patient’s presentation was consistent with a severe case of hydralazine-induced AAV. Diagnosis was confirmed in this case given the evidence of necrotizing granulomatous inflammation and vasculitis in affected tissues, with positive ANCA findings, positive anti-histone antibodies, elevated anti-MPO antibodies, hematuria, proteinuria, and kidney biopsy findings in the setting of hydralazine use [7].

Typical presentations are often nonspecific, with constitutional symptoms such as fatigue, weight loss, and fever. Multiple organ systems may be involved, most commonly the kidneys, where hematuria, proteinuria, and rapidly progressive glomerulonephritis are characteristic findings. Pulmonary manifestations occur in approximately 30-40% of patients and may include dyspnea and pulmonary infiltrates [8]. In this case, the patient’s initial chest imaging findings were attributed to pneumonia, which may have masked the underlying vasculitic process.

Pulmonary hemorrhage, although less common, occurs in up to 10% of patients with hydralazine-induced AAV and often correlates with more severe disease [9]. The patient’s presentation with hemoptysis was consistent with this finding. However, hemoptysis is a nonspecific symptom more commonly associated with other conditions, such as pulmonary embolism or severe pneumonia, causing alveolar-capillary membrane and bronchial mucosal injury [10]. Given this patient’s ongoing anticoagulation for a prior DVT, pulmonary embolism was considered but deemed less likely.

The patient’s renal biopsy demonstrated pauci-immune necrotizing glomerulonephritis with crescents, confirming severe renal involvement. In such cases, immunosuppressive therapy with cyclophosphamide or rituximab in combination with steroids is indicated. Plasmapheresis may be beneficial for patients with pulmonary hemorrhage or refractory disease.

Long-term outcomes for hydralazine-induced AAV are generally similar to those of primary AAV, assuming that hydralazine is promptly discontinued and immunosuppression is initiated [11]. However, advanced kidney injury at presentation or delays in diagnosis can result in poor outcomes, including end-stage renal disease or death, in up to 37% of patients [6]. This case underscores the importance of early recognition, multidisciplinary management, frequent monitoring, and close follow-up for patients with suspected drug-induced AAV.

## Conclusions

This case describes a female patient presenting with hemoptysis and dyspnea in the context of recent hydralazine use, whose diagnosis was complicated by underlying pneumonia and gastrointestinal bleeding. Her hospital course resulted in a brief decline requiring intensive care in the setting of intubation and major hemorrhage. Her diagnosis of hydralazine-induced AAV involving both the renal and pulmonary systems was confirmed through dual ANCA positivity and renal biopsy findings in the setting of hydralazine usage. Rapid diagnosis and initiation of immunosuppressive therapy were essential to her recovery, as delayed treatment is associated with poor outcomes. While the patient recovered, ongoing close outpatient monitoring and continued immunosuppression remain essential to prevent relapse. This case highlights that hydralazine-induced AAV, while rare, can present with severe, life-threatening complications resulting in rapid decline that require rapid diagnosis and multidisciplinary management.
